# Seroprevalence of brucellosis and Q fever infections amongst pastoralists and their cattle herds in Sokoto State, Nigeria

**DOI:** 10.1371/journal.pone.0254530

**Published:** 2021-07-20

**Authors:** Simeon Cadmus, Samson Polycarp Salam, Hezekiah Kehinde Adesokan, Kelechi Akporube, Fiyinfoluwa Ola-Daniel, Emmanuel Jolaoluwa Awosanya

**Affiliations:** 1 Department of Veterinary Public Health and Preventive Medicine, University of Ibadan, Ibadan, Nigeria; 2 Center for Control and Prevention of Zoonoses, Faculty of Veterinary Medicine, University of Ibadan, Ibadan, Nigeria; 3 Nigeria Field Epidemiology and Laboratory Training Program, Abuja, Nigeria; University of Liverpool, KENYA

## Abstract

Brucellosis and Q fever are neglected zoonoses of global health importance, with unknown true prevalence in occupationally vulnerable settings, partly due to misdiagnosis for other febrile conditions and poor access to primary health care. We examined the seroprevalence of these diseases and associated factors amongst pastoralists and their cattle in Sokoto State, a hub of cattle and pastoral populations in Nigeria. Serum samples randomly collected from 137 pastoralists and 366 cattle from 27 herds in three selected Local Government Areas (LGAs) in the state were analysed for antibodies to *Brucella abortus* using Rose Bengal Plate Test (RBT) and competitive Enzyme-Linked Immunosorbent Assay (cELISA) as well as antibodies to *Coxiella burnetti* using indirect ELISA. Consenting pastoralists’ knowledge, perception and practices about the diseases were assessed using a semi-structured questionnaire. Data were analysed using descriptive statistics and bivariate analysis at p ≤ 0.05 level of significance. Brucellosis adjusted individual seroprevalence were 0.83% (95%CI: 0.04–4.59%) and 0% among pastoralists; 2.28% (95%CI: 1.16–4.43%) and 5.70% (95%CI: 3.68–8.74%) in cattle by RBT and cELISA, respectively. Adjusted herd-level seroprevalence for brucellosis were 23.20% (95%CI: 11.07–42.54%) and 42.00% (95%CI: 25.27–61.11%) by RBT and cELISA, respectively. For Q fever, higher seroprevalence of 62.57% (95%CI: 54.04–70.46%) and 2.98% (95%CI: 1.57–5.58%) were recorded amongst the pastoralists and their cattle, respectively. with adjusted herd-level seroprevalence of 40.36% (95%CI: 22.57–63.17%). The LGAs of sampling were significantly (OR: 0.2; 95%CI: 0.02–1.00) associated with Q fever infection, though marginal. The majority of the pastoralists had poor knowledge, perception and practices towards the diseases. This is the first study establishing the presence of brucellosis and Q fever at the human-animal interface in Sokoto State, Nigeria. The pastoralists’ poor knowledge, perception and practices about these diseases are worrisome and are important factors for consideration in disease control.

## Introduction

Brucellosis and Q fever are among the Neglected Zoonotic Diseases (NZD) which are largely eliminated in developed countries, but under-diagnosed and under-reported in developing countries [1]. Human brucellosis and Q fever are important causes of fever of unknown origin in both health care setting and community level [2]. The incidence of human brucellosis may be related to the prevalence in animals, especially in settings with poor disease control in animals [3]. Brucellosis could be acquired in individuals through consumption of contaminated unpasteurised milk and milk products as well as through breaks in the skin following direct contact with infected animal tissues or blood or their discharges [4]. Prevalence rates of brucellosis among livestock workers across different parts of the world, particularly in developing nations, ranging from less than 1.0% to as high as 60.0% [5, 6]. For instance, in parts of pastoral and agro-pastoral settlements in northern Tanzania, the prevalence of between 0.70% and 13.0% of the populace have been reported. Similarly, Regassa *et al*. [7] reported that 60.60% of brucella-positive patients in Ethiopia were pastoralists. In Nigeria, the seroprevalence of 31.82% and 7.60% among livestock workers and hospital patients in the southwestern and north-central parts of the nation, respectively, have been reported [8, 9].

On the other hand, Q fever caused by the rickettsia-like bacterium *Coxiella burnetii* [10] occurs worldwide except for New Zealand [11]. It is endemic, both in animals and humans in sub-Saharan Africa [12, 13] and domesticated animals, including cows, sheep and goats are among the primary sources of human infections [10]. In Nigeria, a report on the seroepidemiological investigation of Q fever uncovered a high prevalence of 44% among hospitalized patients [14].

The use of the Rose Bengal Plate Test (RBT) and Competitive Enzyme-Linked Immunosorbent Assay (cELISA) for the diagnosis of brucellosis [15, 16] has been earlier reported. The sensitivity and specificity of RBT for diagnosis of brucellosis were 95.8% and 100% in cattle [17], and 87.5% and 100% in humans [18, 19], respectively. For the cELISA, the sensitivity and specificity were 97.1% and 100% in cattle [20], respectively. The use of Indirect IgG Phase II ELISA for Q fever diagnosis has been reported ((Dean *et al*., [21] with sensitivity and specificity being 82.6% and 100% in cattle [22], and 98% and 100% in humans [23], respectively. Both brucellosis and Q fever are usually misdiagnosed due to the associated febrile symptoms in most health facilities in Nigeria characterized with inadequate testing capacity, thus resulting in long debilitating illness and under-reporting of the diseases [24, 25]. Again, the pathogens of these diseases are regarded as potential bioterrorism agents [26], and information on the prevalence of the infections in sub-Saharan Africa is scanty [27]. The pastoralists are known for practices that could enhance disease transmission; yet, data on the prevalence of these diseases are lacking especially in northern Nigeria. This region is characterized by a human-animal interface that could facilitate disease transmission. Hence, this study was designed to determine the seroprevalence of brucellosis and Q fever amongst pastoralists and their cattle as well as assess the pastoralists’ knowledge, perception and practices about the diseases in Sokoto State, northwestern Nigeria.

## Materials and methods

### Ethics statement

Ethical clearance for the study was obtained from the Health Research Ethics Committee of Sokoto State Ministry of Health (Approved Number: SMH/1580/V.IV SKHREC/02/019) and the Animal Care Use and Research Ethics Committee of the University of Ibadan, Nigeria. Oral consent was also obtained from willing participants and parents in the case of minors after the purpose of the study had been explained to them. The collection of human blood was by medical health personnel while considering the participants’ welfare. Blood collection from cattle was done by a veterinarian. Animal welfare was considered as minimal time used to restrain them thereby reducing stress and suffering. After proper restraint by the cattle handlers, 5mLs of blood was collected from each cattle and 2-3mls from pastoralists. Blood samples were collected from those willing to participate in the study.

### Study area

**Fig 1:**
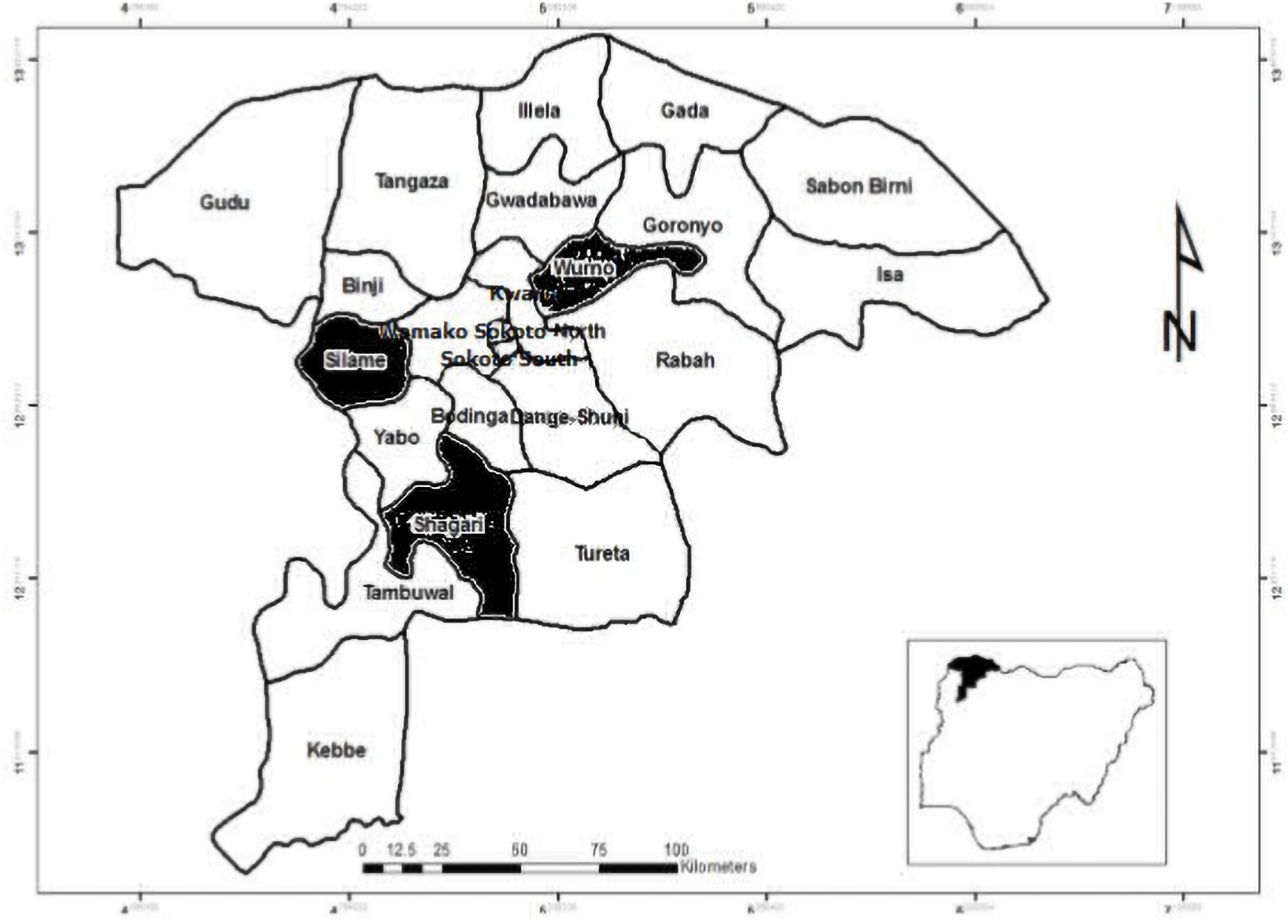
Map of Sokoto State showing study locations (Wurno, Silame and Shagari shaded in black); with Nigeria and Africa as insets.

The study was conducted in three Local Government Areas (LGAs) (Wurno, Silame and Shagari) in Sokoto State, northwestern Nigeria (Fig 1).

The state has a total landmass area of 25,973km^2^ divided into three senatorial districts namely; Sokoto East, Sokoto North and Sokoto South. Its northern boundary is shared with the Niger Republic while to the west, Benin Republic. It had a human population of 3,696,999 based on the 2006 general census [28] with an estimated human population of 4,602,298 projected for 2013. The main economic activities in the area are farming and cattle rearing and the areas across the three senatorial zones constitute an important hub of pastoralists in the state; hence, a veritable point for disease surveillance and control for the targeted diseases. Sokoto has 23 LGAs, with eight LGAs in Sokoto East and Sokoto North each and seven in Sokoto South senatorial district. Wurno, Silame and Shagari LGAs were randomly selected by balloting from LGAs in Sokoto East, Sokoto North and Sokoto South senatorial districts, respectively.

### Study design, sampling technique and eligibility criteria

A cross-sectional study was conducted amongst cattle herds and pastoralists in three randomly selected LGAs in Sokoto State. The formula $n=Z^{2}p\left(1-p\right)*D/E^{2}$ by Thrusfield [29] was used to determine the number of herds required (122 herds); where Z (1.96) is the reliability coefficient at 95% confidence level; p is the expected prevalence at 50%; D (1.27) is the design effect and E is the precision at 10%. D was obtained using the formula $D=1+\left(b-1\right)\rho$ by Bennett *et al*. [29] where b (4) is the average number of samples per herd (about 20% of cattle per herd), ρ (0.09) is the intra-cluster correlation coefficient from previous studies [30]. There are 610 available cattle herds, of which 122 were simple randomly selected. However, only 27 herds (22.1%) consented to participate. We ended up sampling 50–100% of each cattle herd resulting in a total of 366 cattle. The herds had a range of 10 to 29 cattle per herd. Every willing and consenting pastoralist working with the participating cattle herds was also screened, which gave a total of 137 pastoralists.

### Sample collection procedure

The areas for blood collection in both cattle and humans were disinfected using methylated spirit before sampling. Five mls of the sample was collected by a veterinarian from the jugular vein of selected cattle and 2-3mls by medical personnel from each consenting pastoralist using sterile needles and syringes into plain vacutainers. The samples were labelled appropriately using codes to differentiate those of pastoralists from their cattle and transported in separate transport coolers containing ice-packs to the Tuberculosis and Brucellosis Laboratories of the Department of Veterinary Public Health and Preventive Medicine, University of Ibadan, Ibadan. To obtain sera, the samples were centrifuged at 3,000 rpm for 10 mins and stored in cryo-tubes at -20°C until they were assayed.

### Questionnaire administration

A pre-tested semi-structured 22-item questionnaire was interviewer-administered to consenting pastoralists in the study area to assess their knowledge, perception and practice on brucellosis and Q fever. Pre-testing of the questionnaire was done among 10 Fulani pastoralists in Ibarapa Area of Oyo State, with similar characteristics, but not included in the study. The questionnaire was written in English and translated into the participant’s local language (Hausa). It comprised four sections, including basic data relating to demography (age, gender, religion, educational status, residential area, length of time spent in pastoralism), as well as sections on knowledge (10 questions), perception (4 questions) and practice (8 questions) related to brucellosis and Q fever. Only 56 (40.88%) of the 137 pastoralists participated in the questionnaire survey; the others declined due to apprehension associated with security issues.

### Laboratory analysis

#### Brucellosis testing

The Rose Bengal Plate test (RBT) and Competitive Enzyme-Linked Immunosorbent assay (cELISA) were used for the diagnosis of brucellosis.

The RBT was carried out to detect the absence or presence of the agglutination process [31, 32]. Briefly, equal volumes (30 μL) of *B*. *abortus* antigen and test serum were mixed thoroughly on the Rose Bengal plate using a wooden applicator and the plate was rocked for 4 minutes. The appearance of agglutination within 1 minute was scored 2+ (++), whilst any agglutination between 1 and 4 minutes was scored 1+ (+). The absence of agglutination within 4 minutes of rocking was regarded as negative (−). Also, for the cELISA, the reagents in the kit were reconstituted and the test carried out following the protocol earlier described by MacMillan *et al*. [33]. Briefly, the conjugate solution was prepared and diluted to working strength. Approximately, 20 μl each of the test serum was added per well. Columns 11 and 12 were left as controls. Then, 20 μl of the negative control was added to wells A11, A12, B11, B12, C11 and C12, while 20μl of the positive control was added to wells F11, F12, G11, G12, H11 and H12. The remaining wells of columns 11 and 12 that have no serum added, act as the conjugate controls. Thereafter, 100μl of the prepared conjugate solution was dispensed into all wells giving a final serum dilution of 1/6. The plate was vigorously shaken on the microtitre plate shaker for two minutes. The plate was covered with the lid and incubated at room temperature (21°C ± 6 °C) for 30 minutes on a rotary shaker at 160 revs/min. The contents of the plate were shaken out and rinsed five times with washing solution (one ampoule of Na2HPO4 and 1 ml of Tween 20 to 10 litres of distilled water) and then thoroughly dried by tapping on an absorbent paper towel. The microplate reader was switched on and allowed the unit to be stabilized for 10 minutes. The substrate and chromogen solutions were prepared immediately before use, of which 100 μl were added to each well. Then, the plate was left at room temperature for a minimum of 12 minutes. Up to 100 μl of stopping solution was then added to all wells to slow the reaction. Condensation of the bottom of the plate was removed with an absorbent towel and then the plate was read at 450 nm.

Positive samples had a clear appearance whereas negative samples appeared orange in colour. The optical density (OD) was measured at 450nm using a microplate ELISA reader and cut-offs for the titres was determined based on the manufacturer’s instructions.

#### Q fever testing

The Phase II Indirect Enzyme-Linked Immunosorbent assay (iELISA) was used for the diagnosis of Q fever infection. The reagents in the kit were prepared and the tests carried out according to the manufacturer’s instructions. Each plate containing 96 wells was pre-coated with *Coxiella burnetti* phase I and II antigens. The reagents in the kit included conjugate, wash concentrate, substrate solution, dilution buffers, control sera and stopping solution. Before the use of the reagents, each one was reconstituted according to the manufacturer’s instruction. Briefly, 90 ul of dilution buffer was dispensed to each well. Thereafter, 20 ul of the positive control was added to two wells and same was done for the negative control into another two wells. Then, 10 ul of each sample to be tested was dispensed into the remaining wells. Incubation of the plate was done at 21°C. The content of the plate was then emptied and the plate washed three times with wash solution. Immediately, 100 ul of the conjugate was dispensed into each well. The plate was then incubated for another 30 minutes, after which it was then re-emptied and re-washed thrice with 300ul of wash solution. Finally, 100 ul of the substrate (Urea hydrogen peroxide) and chromogen mixture were added to each well and the plate left for 15 minutes at room temperature. The reaction was stopped by the addition of 100 ul of stopping solution (citric acid solution) into all wells. In the presence of antibodies (positive), a blue solution appeared which became yellow after the addition of the stop solution. In the absence of antibodies (negative), no colouration appeared. The optical densities (OD) were then measured at 450 nm in a microplate ELISA reader and cut-offs for the titres were determined based on the manufacturer’s instructions.

### Data analysis

The data obtained from serology and the questionnaire were entered into a spreadsheet and analyzed using SPSS (version 20.0). Counts, frequencies and percentages were obtained for qualitative variables, while summary statistics were determined for quantitative variables. Apparent prevalence at 95% Wilson’s confidence limit was determined by dividing the positive samples by the total number of samples screened. The apparent prevalence was adjusted using the Rogan-Gladen estimator to determine the true prevalence at 95% Blaker’s exact confidence limit with the aid of Epitool online software available at https://epitools.ausvet.com.au/trueprevalence. A positive herd (either for brucellosis or Q fever) was any herd having at least one animal with a positive test result.

Knowledge level was determined using a scale of 10 with each question having equal weight; a score of 5 and above adjudged as good using 50^th^ percentile; while perception and practice levels were on the scales of 4 and 8 with scores of ≥2 and ≥4, respectively adjudged as good. Association between serological tests (outcome variable) and categorical or independent variables including demographics, knowledge, perception and practice levels were determined by calculating the odds ratio using a standard cross-tabulation (2x2 table). The level of statistical significance in the association between the outcome variable and other independent variables was analyzed using the Fisher exact test. Values of α ≤ 0.05 were considered statistically significant.

## Results

### Questionnaire survey

Of the 137 pastoralists who presented themselves for blood sampling, only 56 (40.88%) respondents consented to be interviewed, belonging to Shagari (57.14%, n = 32), Silame (28.57%, n = 16) and Wurno (14.29%, n = 8) LGAs (Table 1). The median age of the respondents was 26 years (Range: 10–56 years). All participants were Muslims, with 95.0% being males. The majority (71.00%) of the respondents did not have any formal education, with only 2% having tertiary education. Up to 27 (48.21%) had been involved in pastoralism for less than or equal to 19 years (Table 1).

**Table 1:** Socio-demographic variables of pastoralists who participated in the questionnaire survey (n = 56) in Sokoto State, Nigeria.

Demographic variable	Freq (%) n = 56
**Age**	
≤ 19	14 (25.0)
20–30	18 (32.2)
31–40	12 (21.4)
> 40	12 (21.4)
**LGA**	
Wurno	8 (14.3)
Silame	16 (28.6)
Shagari	32 (57.1)
**Gender**	
Male	53 (94.6)
Female	3 (5.4)
**Years of Practice**	
≤19	27 (48.2)
20–30	18 (32.1)
31–40	8 (14.3)
>40	3 (5.4)
**Educational qualification**	
Non formal	40 (71.4)
Primary	7 (12.5)
Secondary	8 (14.3)
Tertiary	1 (1.8)

Almost 93.0% of the respondents had poor knowledge of the definition, clinical signs and prevention of brucellosis and Q fever. About 7% believed that people infected with brucellosis and Q fever can be quarantined, 80.4% were indifferent as to whether the infections could be completely cured in an infected human or animal. Again, 8.9% disagreed that a person with suspected brucellosis or Q fever infection was still a potential source of danger to other pastoralists even after treatment. About 89.9% of the respondents had a poor perception of prevention against Q fever. By way of practice, 89.3% of respondents did not consider it important to wear personal protective clothing when working. Similarly, only 1.8% would use protective equipment while attending to their sick herds and 83.9% had never worn protective equipment. The majority of the pastoralists (96.4%) reported not having had contact with aborted fetuses. About 92.9% consume livestock products (milk and meat) and 82.1% had contact with animal urine, faeces (as manure) and hide/skin (trade). Up to 28.6% would not go for a medical checkup while 35.7% each would visit the hospital and traditional healer for treatment if a health problem arose. About 92.9% of the respondents had poor prevention practice against Q fever. However, none of the respondents’ knowledge level, perception or practice was significantly associated with seropositivity to *C*. *burnetii* antibodies.

### Seroprevalence of brucellosis

The apparent and adjusted individual seroprevalence of 2.19% (8/366; 95% CI: 1.11–4.25%) and 2.28% (95% CI: 1.16–4.43%) for *Brucella* infection were obtained in cattle by RBT; 5.54% (19/343; 95% CI: 3.57–8.49%) and 5.70% (95% CI: 3.68–8.74%) using cELISA, respectively (Table 2). The apparent and adjusted herd-level prevalence for brucellosis among cattle were 22.22% (6/27; 95% CI: 10.61–40.76%) and 23.20% (95% CI: 11.07–42.54%) by RBT; 40.74% (11/27; 95% CI: 24.51–59.27%) and 42.00% (95% CI: 25.27–61.11%) using cELISA, respectively (Table 2). Among the pastoralists, the apparent and adjusted seroprevalence for *Brucella* infection using RBT were 0.73% (1/137; 95% CI: 0.13–4.02%) and 0.83% (95% CI: 0.04–4.59%), respectively. None was positive with cELISA (Table 3). Seroprevalence of Q fever.

**Table 2:** Apparent and adjusted individual and herd seroprevalence of brucellosis, Q fever, and co-infection of both diseases in cattle, Sokoto State, Nigeria.

Characteristic	Number positive	Apparent prevalence % (95% CI)	Adjusted prevalence% (95% CI)
**Individual**			
Brucellosis (RBT) n = 366	8	2.19 (1.11–4.25)	2.28 (1.16–4.43)
Brucellosis (c-ELISA) n = 343	19	5.54 (3.57–8.49)	5.70 (3.68–8.74)
Q fever (i-ELISA) n = 366	9	2.46 (1.30–4.61)	2.98 (1.57–5.58)
Coinfection of Brucellosis and Q fever n = 343	3	0.87 (0.30–2.54)	-
**Herd (n = 27)**			
Brucellosis (RBT)	6	22.22 (10.61–40.76)	23.20 (11.07–42.54)
Brucellosis (c-ELISA)	11	40.74 (24.51–59.27)	42.00 (25.27–61.11)
Q fever (i-ELISA)	9	33.33 (18.64–52.18)	40.36 (22.57–63.17)
Coinfection of Brucellosis and Q fever	3	11.11 (3.85–28.06)	**-**

**Table 3:** Apparent and adjusted seroprevalence of brucellosis, Q fever, and co-infection of both diseases among pastoralists in Sokoto State, Nigeria (n = 137).

Characteristic	Number positive	Apparent prevalence % (95% CI)	Adjusted prevalence % (95% CI)
Brucellosis (RBT)	1	0.73 (0.13–4.02)	0.83 (0.04–4.59)
Brucellosis (c-ELISA)	0	0 (0.00)	-
Q fever (i-ELISA)	84	61.31 (52.96–69.05)	62.57 (54.04–70.46)
Coinfection of Brucellosis and Q fever	1	0.73 (0.13–4.02)	-

The iELISA had an apparent and adjusted individual seroprevalence of 2.46% (9/366; 95% CI: 1.30–4.61%) and 2.98% (95% CI: 1.57–5.58%) for Q fever amongst cattle, respectively. The apparent and adjusted herd-level seroprevalence for Q fever in cattle were 33.33% (9/27; 95% CI: 18.64–52.18%) and 40.36% (95% CI: 22.57–63.17%), respectively (Table 2). Among the pastoralists, the apparent and adjusted seroprevalence for Q fever were 61.31% (84/137; 95% CI: 52.96–69.05%) and 62.57% (95% CI: 54.04–70.46%), respectively (Table 3). Eighty-four of the infected pastoralists were linked to 18 of the 27 cattle herds screened.

#### Brucellosis-Q fever co-infection

Individual-and herd level co-infections with brucellosis and Q fever were 0.87% (3/343; 95% CI: 0.30–2.54%) and 11.11% (3/27; 95% CI: 3.85–28.06%) in cattle, respectively (Table 2). Human co-infection with brucellosis and Q fever was observed in one (0.73%; 95% CI: 0.13–4.02%) of the 137 pastoralists (Table 3).

Pastoralists from Silame LGA were less likely (OR: 0.2; 95% CI: 0.02–1.00) to be seropositive to *Coxiella burnetii* when compared with pastoralists at Shagari LGA; though marginally significant (Table 4). No other demographic variable was associated with seropositivity to *Coxiella burnetii* among the pastoralists (Table 4).

**Table 4:** Association between seropositivity to *Coxiella burnetii* antibodies and pastoralists’ demographic variables in Sokoto State, Nigeria.

Demographic variable	Q fever status (iELISA)		OR (95% CI)	P-value
	Positive	Negative		
	n = 47 (%)	n = 9 (%)		
**Age**				
≤ 19	13 (92.86)	1 (7.14)	2.5 (0.1–165.0)	0.88
20–30	14 (77.78)	4 (22.22)	0.7 (0.1–6.1)	1.00
31–40	10 (83.33)	2 (16.67)	1 (0.1–16.4)	1.00
> 40	10 (83.33)	2 (16.67)	1	
**LGA**				
Wurno	8 (100.00)	0 (0.00)	∞ (0.1–∞)	1.00
Silame	10 (62.5)	6 (37.5)	0.2 (0.02–1.0)	0.04*
Shagari	29 (90.62)	3 (9.38)	1	
**Gender**				
Male	44 (83.02)	9 (16.98)	0.0 (0.0–13.4)	1.00
Female	3 (100.00)	0 (0.00)		
**Knowledge**				
Poor	43 (82.69)	9 (17.31)	0.0 (0.0–8.4)	0.97
Good	4 (100.00)	0 (0.00)		
**Years of Practice**				
≤19	24 (88.89)	3 (11.11)	3.7 (0.05–95.9)	0.36
20–30	15 (83.33)	3 (16.67)	2.4 (0.03–61.9)	0.49
31–40	6 (75.00)	2 (25.00)	1.4 (0.02–46.9)	1.00
>40	2 (66.67)	1 (33.33)	1	
**Educational qualification**				
Non formal	32 (80.00)	8 (20.00)	1	
Formal	15 (85.71)	1 (14.29)	3.7 (0.4–177.3)	0.40

Note:

*Significant at P < 0.05;

∞ = undefined.

## Discussion

This present study is the first to investigate brucellosis and Q fever in linked animal and human populations in Sokoto State, Nigeria. Simultaneous assessment of the exposure of humans and animals provides a more complete epidemiological picture and deepens our understanding of infection patterns at the animal-human interface. Hence, our findings are not only relevant to the state, but also neighbouring countries in West Africa, given the frequent trans-border movement of livestock and humans within that region and the study area.

A previous report indicated a high seroprevalence of brucellosis in the pastoral area [34]. However, this study obtained low apparent and adjusted seroprevalence of brucellosis in humans (0.73% and 0.83% for RBT; 0% and 0% for cELISA, respectively) and cattle (2.19% and 2.28% for RBT; 5.54% and 5.70% for cELISA, respectively). The low adjusted prevalence of brucellosis in humans in this study is similar to the report of Dean *et al*. [21] who reported a 1.0% seroprevalence among humans in livestock villages in Togo. One possible explanation for this present finding could be due to the observation that the majority of the animals were apparently healthy. This might lower the risk of exposure of the pastoralists to brucellosis infection. Meanwhile, the fact that 92.9% consume livestock products (milk and meat) and even had contact with animal urine, faeces (as manure) and hide/skin (trade) raises concern about the exposure risks associated with such practices.

The adjusted seroprevalence obtained by cELISA in cattle in this study is similar to that reported in a related study [9] in southwestern Nigeria where trade cattle were reported to have a prevalence of 5.8% for brucellosis. It is however lower than that reported by Adamu *et al*. [35] among cattle in the agro-ecological zone of Kaduna State, northwestern Nigeria where 18.5% and 6.8% were seropositive to *Brucella* antibodies by RBT and cELISA, respectively. Contrary to our results, Junaidu *et al*. [36] reported an overall high seroprevalence of 32.2% for brucellosis using the RBT, Serum Agglutination Test and cELISA methods in a herd of cattle in Sokoto prison farm. This could be due to the poor hygiene and sanitation of the environment in the presence of *Brucella* organism, which could promote intra- and inter-transmission. Aworh *et al*. [37, 38] also reported a high seroprevalence (24.1%) of *Brucella* infection among abattoir workers, but lower seroprevalence (5.6 and 0.5% for RBT and cELISA, respectively) for cattle slaughtered in Abuja abattoir.

In a meta-analysis, data on the seroprevalence of brucellosis in cattle population in sub-Saharan Africa using Rose Bengal diagnostic test as the reference shows a seroprevalence estimated to be 16.2% with a 95% CI ranging from 10.2% to 25.7% [39]. In Uganda, a seroprevalence of 17.0% for human brucellosis was reported in agro-pastoral communities of Kiboga District [40] which is similar to 15.6% prevalence obtained in Namibia among livestock professionals [41]. However, the present findings show lower proportions in both cattle and humans, possibly suggesting geographical differences.

Similarly, Q fever has been described over 60 years ago and is gradually gaining awareness across the world. Recently, the United States of America Centers for Disease Control (CDC) added Q fever to the list of potential bioterrorism agents making it a reportable disease in some countries. Using the iELISA technique in this study, we obtained apparent and adjusted seroprevalence of 2.46% and 2.98% for cattle and 61.31% and 62.57% for humans, respectively. These findings are of concern considering the wide difference between the adjusted seroprevalence in the two populations. Could it be that there’s a non-virulent strain of *C*. *burnetti* circulating among humans in the study area or is it that the chronic form in humans is asymptomatic [42]? Although, the major route for spreading Q fever from animals to humans is respiratory by breathing in dust that has been contaminated by infected animal faeces, urine, milk, and birth products that contain *C*. *Brunetti*. A previous report showed that lack of appropriate personal protective clothing and residing on a farm increased one’s odds for *Coxiella* seropositivity [43, 44]. It should be noted that up to 89% of the participants in the present study did not have appropriate protective clothing, which might explain the observed high seroprevalence among the pastoralists.

Furthermore, Blondeau *et al*. [14] had earlier reported 44.0% seroprevalence in human patients hospitalized for a variety of acute medical conditions in Sokoto State. It is also similar to the high serological evidence of exposure to *C*. *burnetti* reported among 100 of 278 humans screened in livestock villages in Togo [21]. It is important to note that 37.5% of our respondents would not go to the hospital when having a medical problem. It is therefore disturbing to note that, we are reporting a higher prevalence in the same state twenty-nine years later probably because of the low priority (neglected zoonosis) given to Q fever infection in policy and under-reporting due to misdiagnosis from the health centres. This high adjusted seroprevalence in humans differs from that of an unpublished work in the Ibarapa Area of Oyo State, southwestern Nigeria where an overall seroprevalence of Q fever in humans was 10.68% among the sampled pastoralists in the three LGAs. This might be due to the cultural practice of cohabiting with cattle, unlike those from southwestern Nigeria who have lesser contact time with cattle due to their secondary occupations. Though infection with *C*. *burnetii* in humans can be asymptomatic, symptomatic infection, known as Q fever, may present as an acute undifferentiated febrile illness which can progress to chronic forms typically in individuals predisposed, due to valvular heart disease or immunodeficiency [45]. Using iELISA in an agro-ecological zone of Kaduna State, Adamu *et al*. [35] reported a seroprevalence of 6.2% for Q fever in cattle which is slightly above our present finding. This could be due to the difference in geographical regions; as their study area was a wetland that differed from the drylands of Sokoto State.

In a meta-analysis on Q fever in Africa [46], the following seroprevalence was authenticated for analysis; Northern sub-region (Egypt: 1991, Humans– 32.0%; 2012, Humans– 16%, Buffalo– 0.0%, Cattle– 13%), Western sub-region (Nigeria: 1983–1984, Cattle—55.0%, Senegal: 2007–2008, Cattle– 4.0%, Ghana: 2012, Cattle– 18.0%, Cote d’Ivoire: 1965, Humans– 5.0%), Middle sub-region (Chad: 1999–2000, Cattle– 4.0%, Humans– 1.0%), Southern sub-region (South Africa, 1985–1986, Cattle– 8.0%) and Eastern sub-region (Tanzania: 1993, Humans– 5.0%). An overview of these results suggests that the infection has been under-investigated especially in humans. Besides, the prevalence is also observed to vary with time and geographical locations. As observed in the present study, the location of the sampling was significantly associated with seropositivity to Q fever.

Furthermore, this study reports a co-infection prevalence of 0.73% and 0.87% for *Brucella* infection and Q fever in humans and cattle, respectively. This is in line with an earlier report which indicated that it is likely that co-infection with brucellosis and Q fever occurs in both animals and humans, exacerbating the pathogenesis and outcomes of these zoonotic infections [35]. This is a matter of serious public health concern because pastoral communities in general often lack access to primary health care facilities which is worsened with the lifestyle of delayed health-care seeking behaviour. Thus, possible intra- and inter-transmission of infection could be enhanced. This concern is further heightened by the findings of this present study whereby the pastoralists engaged in activities and practices that could enhance disease transmission at the human-animal interface, including consumption of unpasteurized milk, close and unguarded contacts with cattle as well as handling of potentially contaminated animal materials.

Besides, the study reveals poor knowledge, perception and practices regarding brucellosis and Q fever amongst the pastoralists in the study area. Occupationally exposed individuals have been previously shown to have poor knowledge of zoonoses [47]. As observed in this study, 89.3% of the pastoralists did not agree that it’s important to use protective equipment while only 1.8% had ever used one or more protective equipment. Such a practice might expose the pastoralists to zoonotic infections especially when handling infected cattle population where hygiene levels are also poor. Again, only a little above one-third (35.7%) of the pastoralists in the state agreed that medical checkup was important to a healthy life and would visit the hospitals for treatment when they have health problems. This is critical because brucellosis in humans has a wide range of clinical signs resulting in various diagnostic difficulties as it resembles other febrile conditions including malaria, typhoid, and rheumatic fever [48, 49].

Our findings notwithstanding, this study had some limitations. One, due to the prevailing political insurrection in the state as at the time of sampling, only a limited number of herds and pastoralists were accessed. Higher proportions of herds would have given better insights into the epidemiology of the diseases in the study area. Two, a lower proportion of the sampled pastoralists participated in the questionnaire survey which limited the robustness of inferences that could have been drawn from the study. Notwithstanding, most pastoralist communities especially in the study area have similar herd and pastoralist characteristics. Our findings could therefore be representative of the baseline insight into the seroprevalence of brucellosis and Q fever in the area.

This is the first study of brucellosis and Q fever in linked human and animal populations in Sokoto State, Nigeria, providing baseline data for the country. The study shows that *Brucella* spp and *C*. *burnetii* are circulating in both human and cattle populations in the study area. Although the seroprevalence of brucellosis observed amongst pastoralists in the state is low compared to other places, the disease remains endemic in cattle. High seroprevalence of Q fever antibodies amongst the pastoralists further reiterates the disease as under-reported and neglected. The study further revealed poor levels of knowledge, perception and practice on the two diseases amongst the pastoral communities in the study area. Since diagnosing and treating zoonotic diseases in malaria-endemic countries is particularly challenging, the screening of fever cases for differential diagnoses, including brucellosis and Q fever, should be carried out to determine their role in febrile illness. Routine screening of cattle herds for brucellosis and Q fever should be included in the zoonoses control programme in Nigeria to limit the infections among the cattle population as well as prevent spread to humans. Besides, there is a need to step up enlightenment campaigns against practices that could enhance the transmission of zoonotic infections among pastoralists. Further studies on the isolation and characterization of the *Brucella* and *Coxiella* organisms are also recommended.
